# Are 10-Year-Old Imaging Tests Likely to Be Useful or Can They Be Purged?

**DOI:** 10.1007/s10278-025-01721-1

**Published:** 2025-10-16

**Authors:** Annabelle K. Gaskin, Wayne L. Dell, Alan H. Matsumoto, Cree M. Gaskin

**Affiliations:** 1 College of Liberal Arts and Sciences, University of Virginia, 853 West Main St, Apt. #533, Charlottesville, VA 22903 USA; 2 Department of Radiology, Mass General Brigham, Boston, MA USA; 3 Department of Radiology and Medical Imaging, University of Virginia Health System, Charlottesville, VA USA

**Keywords:** Image storage, Records retention, PACS management, Records management

## Abstract

Many healthcare institutions have large archives of digital medical images. A shared dilemma is whether to purge or continue to store older radiology exams. We sought to determine if our institution’s imaging exams > 10 years old were likely to be used for clinical purposes or if they could be deleted from storage without potential lost benefit to our patients. In compliance with our institutional review board, our institutional PACS database was queried to evaluate if old exams had been opened in the last 2 years. We analyzed by modality, body part, and subspecialty reading division. We performed a retrospective chart review of a random sampling (*n* = 100) of the identified studies to validate the assumption that many of the loaded studies were likely to have been viewed for clinical purposes. We found that over the recent past 2-year interval, our providers accessed 201,000 of 4 million (5%) imaging studies which were > 10 years of age at the time of access. We found evidence, some of it definitive, validating that many of these were viewed for clinical purposes. We conclude that if imaging studies > 10 years of age are readily available, they are commonly called upon to potentially contribute to medical decision-making. This potential value to the patient should be considered when determining whether to prune older archived imaging studies.

## Introduction

Many healthcare institutions have accumulated massive archives of digital imaging studies, including numerous older exams greater than 10 years of age [1, 2]. In addition to privacy and security concerns, these exams are collectively associated with storage, migration, and management costs [1–5]. Historical imaging records could be purged if no longer clinically relevant and within variable institutional, state, and federal guidelines for retention.

When interpreting new clinical imaging tests, it is a well-established standard of care to compare them with relevant prior exams when “appropriate and available” [6]. At what point are older exams no longer likely to be helpful? Unfortunately, there is particularly limited evidence within the peer-reviewed literature to answer this *medical* question. This is especially true during this era of digital imaging when even very old clinical exams can be made available in an instant. Anecdotally, the lack of evidence around the medical utility of very old exams has led to the common practice of indefinite image retention or, in some cases, arbitrary deletion.

One additional variable is that state governments can have records management programs with disposition requirements that direct state institutions like ours to delete their records, including digital medical images, on a schedule [7, 8]. For example, under Virginia’s General Schedule GS-120 for Records Retention and Disposition, effective 2022, digital medical images at Virginia state institutions are to be deleted after 10 years [7, 8]. This deletion specifically applies to “radiology tests including x-rays, computerized tomography (CT) scans, magnetic resonance images (MRI), and ultrasounds” [8]. Further, the policy states that simple deletion of medical images is not sufficient and that it is necessary to overwrite the data or destroy the actual storage media itself [8]. The driving forces behind this policy seem to be concerns around efficiency, privacy, and security, while not citing any medical basis [8].

Our academic health system had long deferred a decision on how many years to store digital medical images prior to deletion, largely due to a lack of guidelines, the manual nature of careful deletion, and the complexity of retention requirements, which we discuss later in this manuscript. More recently, however, our state records disposition policy forced our leadership to confront the potential deletion of clinical imaging tests older than 10 years in order to achieve compliance [8]. Our providers expressed significant concern with this proposition but could only cite anecdotal experience that these older imaging tests might still be of clinical benefit to our patients.

Given the lack of evidence-based guidelines in the medical literature, we needed to determine if our old exams might still have value in the care of our patients. We sought to determine if our institution’s digital imaging studies greater than 10 years of age are still being used for clinical decision-making. This evidence could inform an institutional decision to delete older medical images or retain them for potential future benefit to our patients. Regardless of whether facing a state disposition policy or not, the decision around when to delete old images is a common shared dilemma, and thus, our evidence may be of use to others.

## Materials and Methods

This retrospective study was performed in compliance with our Institutional Review Board (IRB), the Health Insurance Portability and Accountability Act (HIPAA), and the ethical standards stated in the 1964 Declaration of Helsinki.

Our data was obtained by querying the clinical database of our institution’s enterprise Picture Archive and Communication System (PACS) (Vue PACS, Philips Healthcare Informatics Solutions). We performed a large query of the entire database of the PACS to identify exams which were greater than 10 years of age at the time of the query in November 2023. Looking back 2 years (i.e., 730 days) from then, we queried the event logs of the PACS database for imaging studies that had been loaded for viewing and counted the number that were 10 years or older than their respective load dates. We collected data regarding modality, body part, and subspecialty reading division for further analysis. Due to the heavy nature of the query, it was performed after business hours to reduce load on the clinical system and avoid creating a potential conflict with clinical operations.

At the large scale, we sought a holistic perspective of whether old images were loaded for viewing or not. Old studies being loaded on a later date implies that they were used clinically; however, we also sought to validate this assumption. We performed a retrospective chart review of a random sampling (*n* = 100) of our identified loaded old studies to seek potential evidence that they were used again clinically more than 10 years later. Further, we conferred with other radiologists to better understand their reporting practices when interpreting evidence within the medical record.

We summarized the results based upon likelihood that the old studies were actually viewed for clinical purposes when they were loaded 10 + years later. If a 10 + -year-old study was documented in the medical record as having been viewed on the recent load date (e.g., documented in the comparison field of the report of a new radiology study), then this was considered a “definite yes” for having been used clinically by the radiologist. If the medical record documented a clinical encounter on the recent load date of the old study but there was not a corresponding new study, and the clinical notes discussed the relevant anatomic region, then this was considered a “circumstantial yes” to reflect that there is evidence the images were likely viewed for clinical purposes by the referring provider. Additional categories included “anecdotal mix of yes and no,” “inconclusive,” and “speculate no” in descending order of likelihood that studies were viewed for clinical purposes. These categories are explained further in the “Results” section.

## Results

**Fig. 1:**
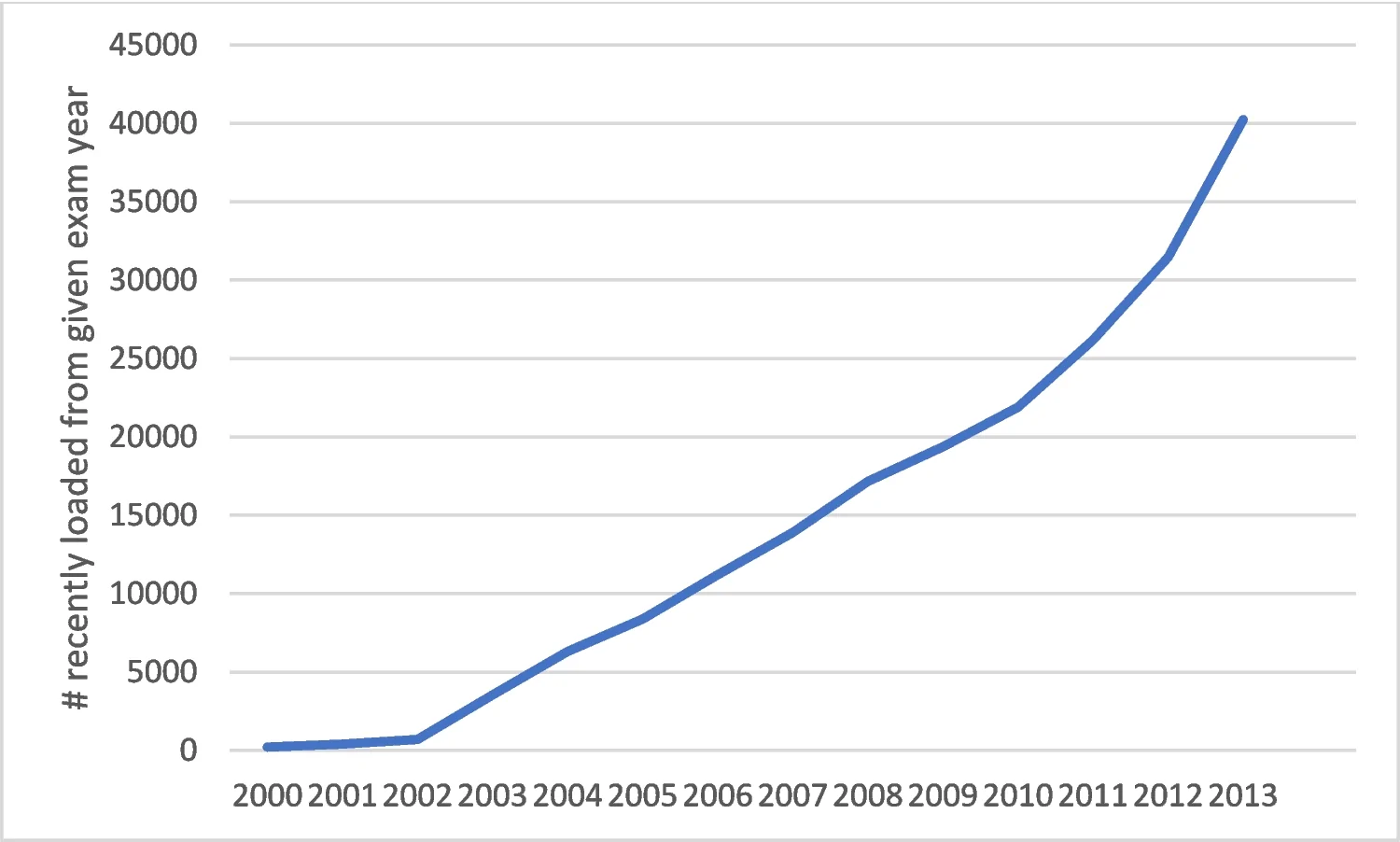
Line chart of the number of old exams recently loaded for viewing which were > 10 years of age at the time of loading. “Recent” was defined as within the last 2 years to support the likelihood of recent clinical relevance. *Y*-axis, number of recently loaded exams from the given old exam year. *X*-axis, year of the old exam which was accessed 10 + years later

**Fig. 2:**
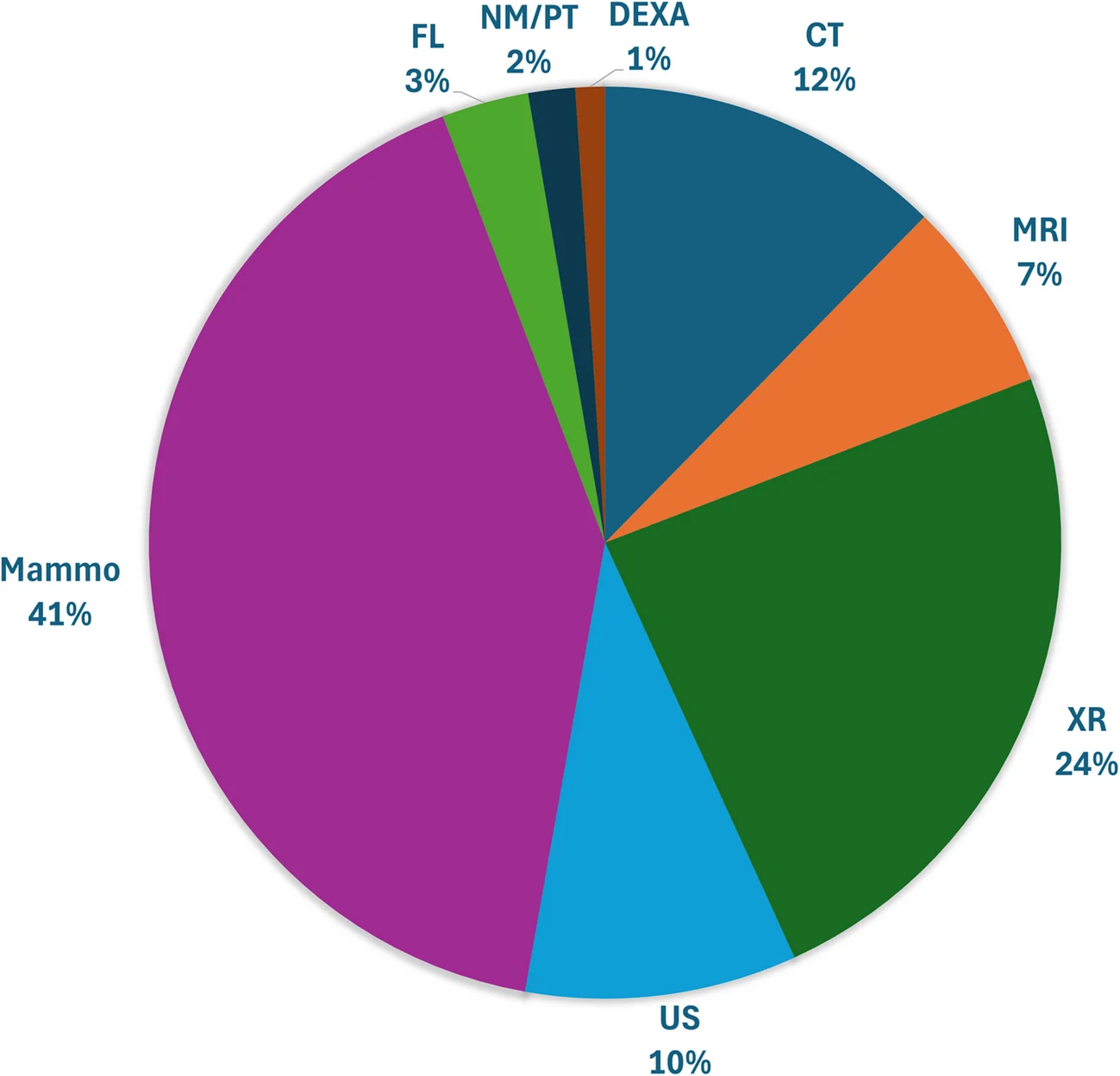
Pie chart showing modality breakdown of old exams recently loaded for viewing which were > 10 years of age at the time of loading. “Recent” was defined as within the last 2 years to support the likelihood of recent clinical relevance. FL, fluoroscopy composed of XA and RF modality types; NM/PT, nuclear medicine including positron emission tomography; XR, radiography composed of CR, DR, and DX modality types; US, ultrasound; CT, computed tomography; MRI, magnetic resonance imaging; DEXA, dual-energy X-ray absorptiometry

**Fig. 3:**
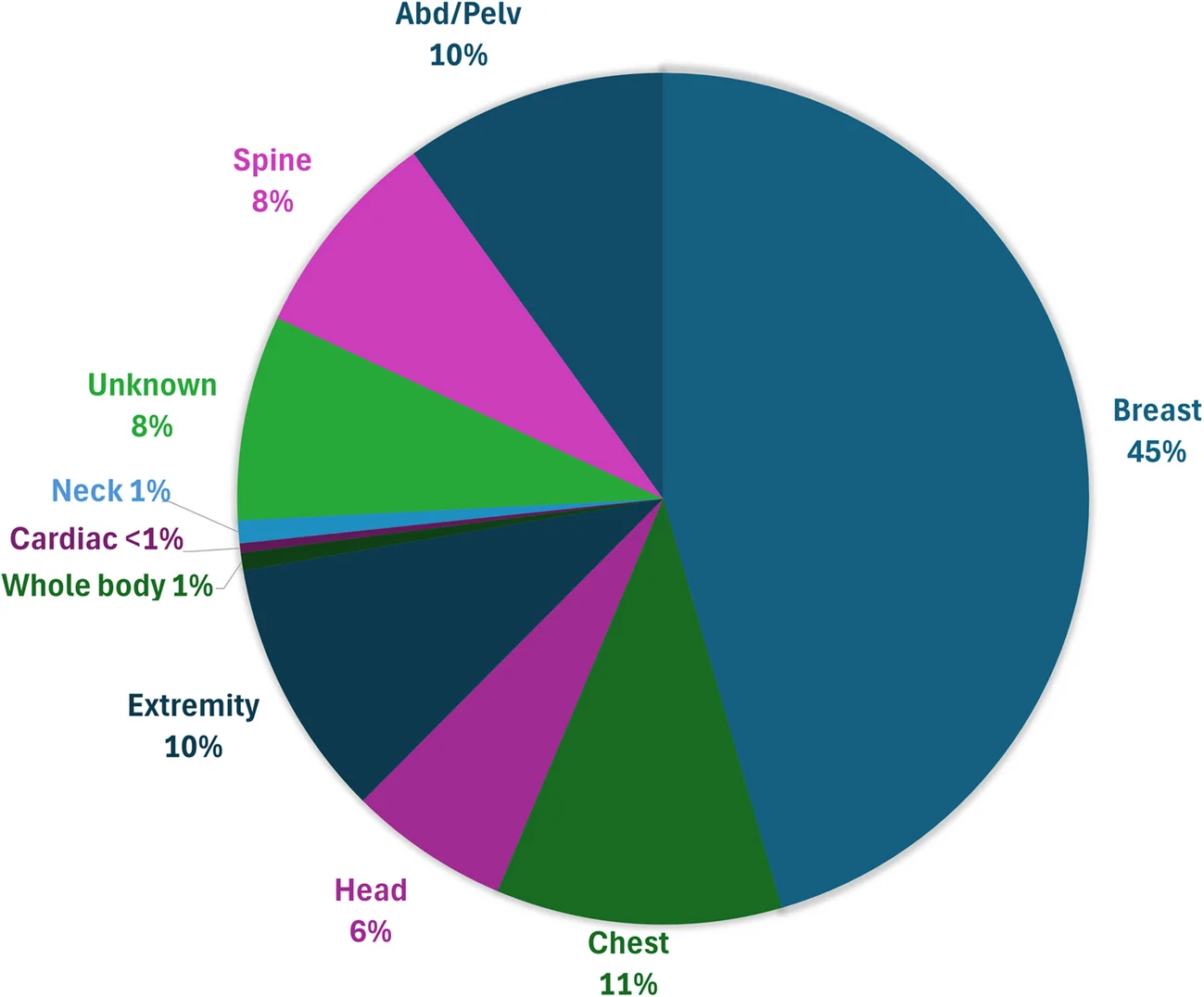
Pie chart showing body part breakdown of old exams recently loaded for viewing which were > 10 years of age at the time of loading. “Recent” was defined as within the last 2 years to support the likelihood of recent clinical relevance

**Fig. 4:**
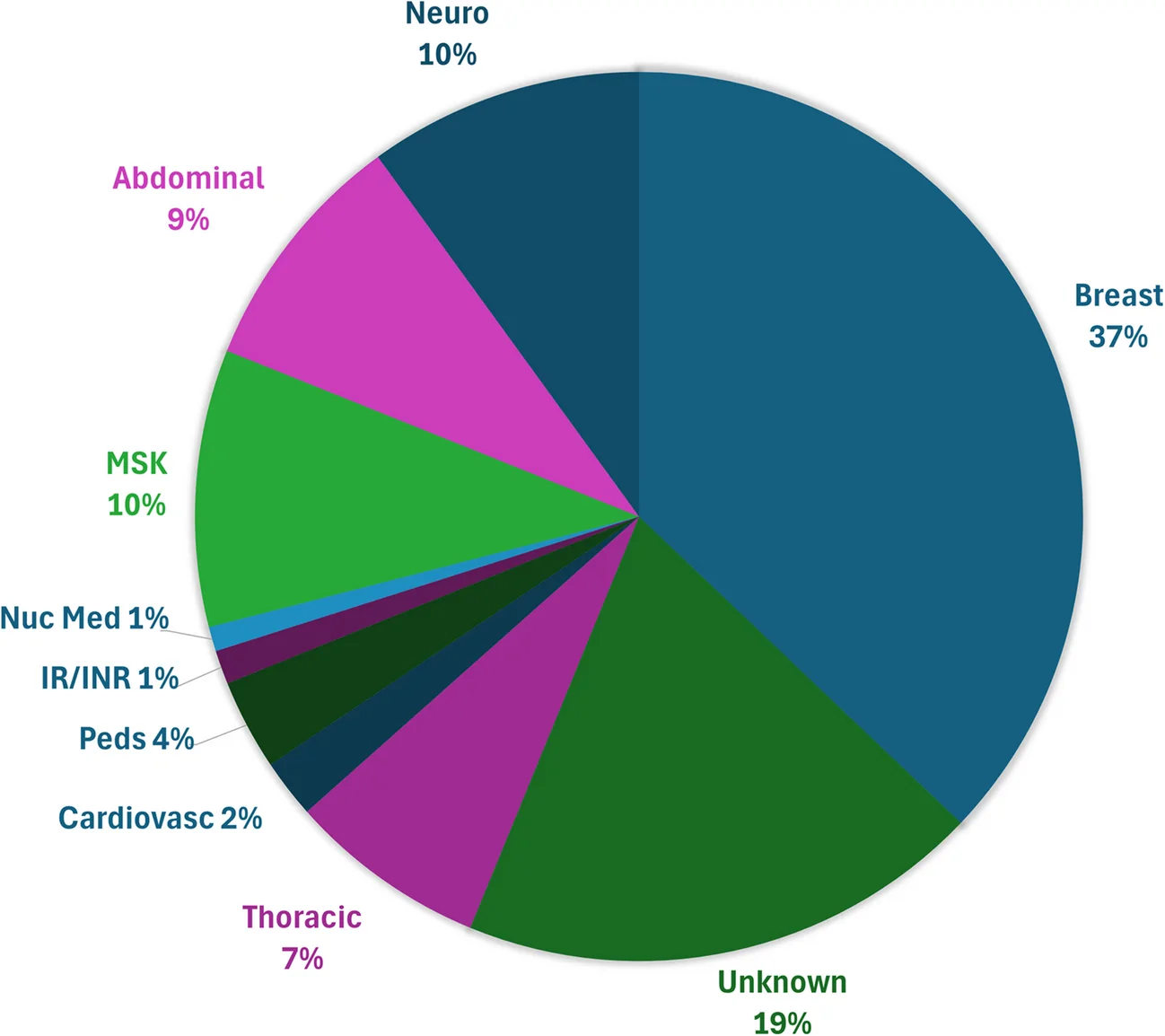
Pie chart showing subspecialty reading division breakdown of old exams recently loaded for viewing which were > 10 years of age at the time of loading. “Recent” was defined as within the last 2 years to support the likelihood of recent clinical relevance. IR/INR, interventional radiology and interventional neuroradiology; Peds, pediatric imaging; Cardiovasc, noninvasive cardiovascular imaging; Nuc Med, nuclear medicine; MSK, musculoskeletal; Neuro, diagnostic neuroradiology

At the time of the query, the institutional PACS database contained 4,341,278 total unique exams *already* greater than 10 years of age, beginning with the exam year of 2000. The PACS database event logs showed that 200,933 studies (5%) were greater than 10 years older than their respective dates of loading for viewing in the previous 2-year period. The overall results are summarized by year in a line chart in Fig. 1. The studies were 41% mammography, 24% radiography, 12% CT, 10% ultrasound, 7% MRI, 3% fluoroscopy, 2% nuclear medicine, and 1% DEXA (Fig. 2). Anatomically, studies were 45% breast, 11% chest, 10% extremities, 10% abdomen/pelvis, 8% spine, 8% unknown, 6% head, and 1% or less each of whole body, neck, or heart (Fig. 3). Studies were spread across subspecialty divisions: 37% breast, 19% unknown, 10% neuro, 10% musculoskeletal, 9% abdominal, 7% thoracic, 4% pediatric, 2% cardiovascular, 1% nuclear medicine, and 1% interventional radiology or interventional neuroradiology (Fig. 4).

**Fig. 5:**
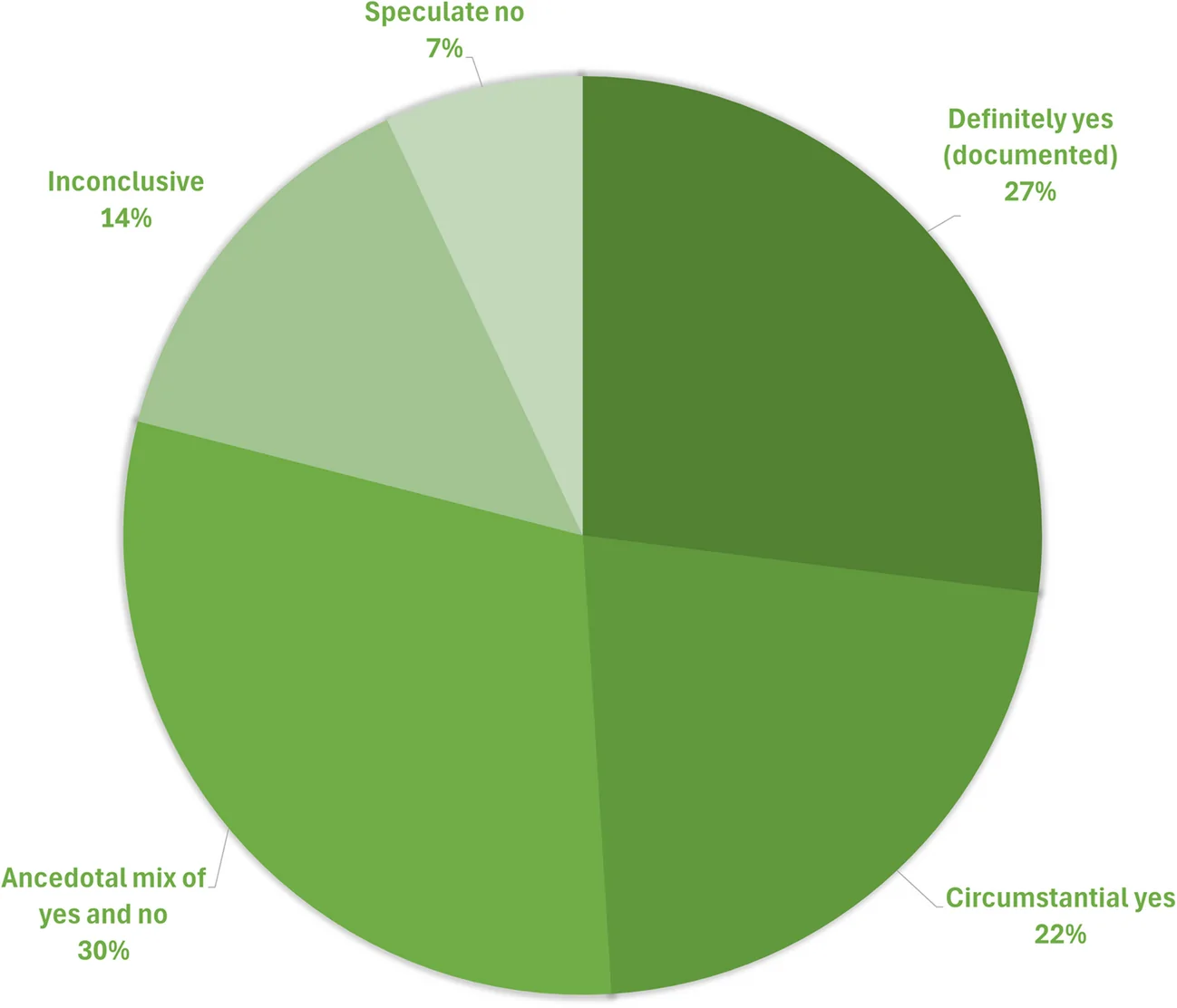
Pie chart showing the relative likelihood that 10 + -year-old exams recently loaded for viewing were actually reviewed for clinical or other purposes. Definitely yes, documentation in new radiology reports of comparison to the old exam. Circumstantial yes, load date corresponded to clinic note discussing conditions of the relevant body part. Anecdotal mix of yes and no, mammographers insisted they use 10 + -year-old exams before recalls or biopsies but do not document it in the report due to system limitations. Inconclusive, no documentation to indicate why the study might have been loaded. Speculate no, group of studies felt likely to have loaded automatically as part of a display protocol though with less clear evidence of utility

The retrospective chart review of a sample (*n* = 100) of the recently loaded studies > 10 years old identified a variety of evidence validating that at least a significant portion of the identified studies were in fact viewed for clinical purposes (Fig. 5). The highest level of evidence (i.e., definite yes) was reached in 27% of the studies, comprised of 13 radiographic exams, 12 CTs, and 2 MRIs, which were documented in the comparison field of the reports of new studies around the load date. The most extreme example was an MRI of the brain where the comparison study was a head CT 22 years earlier. The next level of evidence (i.e., circumstantial yes) was reached in 22% of studies, comprised of 9 radiographic exams, 4 CTs, 4 MRIs, 2 ultrasounds, 1 PET, 1 fluoroscopic exam, and 1 DEXA. These studies corresponded to clinical encounters discussing the relevant anatomic regions (e.g., gout at the foot), but there was not corresponding new imaging.

We noted that 30% of our sample was mammography with load dates corresponding to new mammograms. Every single new mammogram report described comparison to exams up to 5 years old, but none older. We asked our eight breast imaging faculty, who trained at multiple different institutions, if this was evidence that we could safely delete mammograms greater than 10 years old. They were united and emphatic that they commonly use studies this old to avoid callbacks and biopsies, but that their reporting practices and system limitations lead to that not being evident in the report. For example, one wrote, “we frequently use really old studies, especially to look at far posterior and axillary tissue because that’s the one area that’s variably imaged on the mammogram, year for year. I can send you an example of such a case that I read just yesterday and averted a biopsy because the area was best seen on 2010 to 2015 mammograms. She would have had a biopsy if I only looked back until 2018. I would speculate that the reason you might not see older studies listed in the comparison tab on our report is because our comparisons do not automatically populate in the report.” Given the consistent answer, we grouped the old mammographic studies as an “anecdotal mix of yes and no” to reflect that likely some were and some were not used for comparison to new mammograms.

We could not reach a conclusion on 14% of studies, comprised of 5 radiographic studies, 3 MRIs, 2 CTs, 2 ultrasounds, and 2 fluoroscopic studies. The reason for potential loading was simply unclear due to lack of new imaging or new clinical encounters around the load date. The loading may or may not have been used for clinical purposes. Finally, we speculate that 7% of studies were probably not viewed with clinical utility. These comprised 5 radiographic studies, 1 DEXA, and 1 fluoroscopic exam which we believe were likely automatically loaded by a display protocol based upon body part but ultimately were not relevant (e.g., contralateral foot or very clearly compared to more recent prior exams without reason to go deeper in the archive). Still, even some of these could have been used for undocumented clinical reasons like a contralateral comparison.

## Discussion

When interpreting and reporting medical imaging tests, it is a well-established practice standard for radiologists to compare current exams with available relevant prior examinations because this influences image interpretation and related medical decision-making [6, 9, 10]. This is an essential component of a complete evaluation of an imaging test for the optimal benefit of the patient and referring provider. In the context of our current work, “available” is a key word. Stating the obvious, if images are purged, they are not available for comparison even if the patient returns to the same institution they have long entrusted with their care and medical record keeping. For example, a lung nodule found on a recent exam during the workup of a patient with a new diagnosis of cancer may be confidently confirmed to be a benign finding by establishing long-term stability. In this case, a 10-year-old comparison exam which might have been purged under policy is as valuable to this patient as an exam that might have been retained just because it was younger. In fact, the very old exam can be even more powerful than a recent exam by offering greater reassurance of benignity by definitively establishing long-term stability.

The importance of comparing to prior exams has also played out in the courtroom. Juries have found against radiologists in malpractice cases for not aggressively seeking prior exams, which were not immediately available, and for comparing only to the most recent prior exam and not multiple prior exams [9, 10]. The decision by an institution to purge medical imaging studies would most certainly absolve radiologists from comparing to these exams but could still cause potential lost benefit to patients in medical decision-making. It is unclear if this decision to purge old studies could put an institution or its decision-makers at any risk.

While old literature and current practice standards make it clear that comparison is clinically and legally important, there is no clear guideline on when it is safe to delete older studies without surrendering potential benefit to patients [6, 9, 10]. The existing literature on the clinical and legal relevance of old studies tends to focus on the comparison, or lack of comparison, to retained studies, rather than on when it is okay to delete studies. Further, the limited literature tends to be older and focused on radiographs, rather than being from this modern era of digital cross-sectional imaging when old studies are even more complicated and yet available instantly [6, 9, 10]. In our literature search, we struggled to find medical evidence or investigations justifying criteria to purge without lost benefit to patients. Rather, more recent publications on this general subject tend to be commentary or not peer reviewed, and discussions around deletion are from a technical or security perspective rather than on a medical basis [1–5].

We found that approximately 5% of our medical imaging studies older than 10 years of age were still accessed by our providers using the enterprise PACS within just the recent 2-year period. These studies comprised all modalities, anatomical regions, and subspecialty reading divisions. These studies being immediately accessible when desired, in spite of their age, likely contributed to their rate of access and potential clinical utility. We believe it is likely that many of these studies were loaded by radiologists for comparison purposes when interpreting new exams. This loading of prior studies can be manual, but much more often occurs automatically via a display protocol, as designed by the radiologists to their preferences for studies they might find useful, such as by matching body part and modality. Our retrospective chart review did produce definitive evidence of recent direct viewing of old studies for clinical purposes in at least a portion in which the radiologists described comparison to the old studies in their new reports. Further, we obtained anecdotal evidence that sometimes the radiologists compare to very old studies but do not always document them when they are already reporting comparison to more recent intervening studies due to system issues and the additional effort required. The radiologists use these prior exams to make decisions about the significance of interval changes and current imaging findings. Of course, old imaging exams could alternatively have been loaded by clinicians or for purposes of teaching or research. We found significant circumstantial evidence that some of the old studies were loaded by clinicians at the time of clinical encounters discussing the relevant body part. Regardless of who loaded the study, the act of loading is usually intentional, even if automatic by design, for potential impact on clinical decision-making.

We did not specifically collect and sort data regarding which clinical conditions were being evaluated when old comparison studies were loaded; however, based upon the chart review, cancer was among multiple diagnoses. In recent decades, due to advancements in care and earlier diagnosis, there has been an increase in the number of patients living with cancer as a chronic condition who potentially may benefit from the retention of old imaging tests [11, 12]. As a result, having access to imaging studies more than 10 years old may have even greater clinical importance in the future.

Given the potential for lost clinical benefit to patients when purging old studies, we find our results potentially at odds with our state’s current records disposition policy. This policy, intended to improve efficiency and prevent unauthorized disclosure, does not cite medical evidence about the lack of clinical impact to patients when directing our state academic healthcare institution to destroy digital medical images greater than 10 years of age [8]. Meanwhile, in our study, nearly 1 in 20 similarly old exams were potentially called upon for likely clinical use in a practical recent time period. Over the much longer window that we commonly care for our longitudinal patients, this access would likely be even greater and more impactful.

Minimum retention periods for medical imaging are discussed more commonly than disposition requirements in both the medical literature and governmental policies. The joint ACR-AAPM-SIIM Technical Standard for Electronic Practice of Medical Imaging does not establish a standard term for medical image destruction, though it states that facilities must comply with facility, state, and federal regulations regarding minimum periods for medical records retention [13]. Accreditation as a Diagnostic Imaging Center of Excellence by the American College of Radiology requires that medical imaging records are retained for at least 5 years or as required by the state [14]. The Centers for Medicare & Medicaid Services (CMS) requires that medical records are retained 7 years from the date of service [15]. Longer retention requirements for medical images may exist in the setting of research, mammography, pediatrics, and toxic exposures, and these rules can vary by state as well as by type of facility (i.e., a hospital versus a physician’s clinic where imaging is done) [16, 17]. The state-based rules can vary widely and be cumbersome to implement. For example, Legalclarity.org reports that under Massachusetts General Laws Chapter 111, Sect. 70, hospitals must retain medical records for at least 7 years from the last patient encounter. Further, for pediatric patients, the records must be retained for greater than 7 years after the patient turns 18 [18, 19]. In these instances, imaging studies could be significantly older than 7–10 years before they could even be considered for destruction.

Our work is limited in generalizability by involving only one institution. Other institutions might have providers who access old prior studies more or less frequently, though a strength of our study is the large sample size of our automated query. Another limitation is that we primarily identified that a study was loaded in PACS and relied on a small retrospective chart review to validate assumptions that loaded studies were actually viewed for clinical purposes. The loading of an old study implies but does not prove potential clinical utility or impact on patient outcome. In fact, it is possible that some studies were loaded automatically and not even reviewed at all. Still, we did find definitive evidence that some were indeed viewed and reported as a comparison study by radiologists. And there is circumstantial evidence that clinicians also viewed some of the old studies in new clinical encounters. It is not possible to know how many of the loaded cases were actually useful, only that they were considered potentially useful. In some cases, images may have been loaded for non-clinical purposes of no benefit to the patient at all. Finally, our work is not a comprehensive risk–benefit analysis of the long-term storage of medical images from the perspectives of the state, institution, and/or patient.

Whether you are part of a state healthcare institution with state-directed disposition requirements for medical images like ours, or part of a state or private institution without such requirements, we all face practical decisions about whether to purge or retain older records to save costs and protect privacy. Destruction of old medical images would reduce storage and future PACS migration costs but could have unintended clinical consequences for individual patients. Further, these cost savings would be offset and potentially exceeded by new human labor costs as institutions would need to determine which records could be safely deleted from a clinical perspective and which ones, conversely, must be legally retained beyond the regular disposition schedule due to “subpoenas, investigations, or litigation that reasonably may involve [these records]” [8]. Our state policy acknowledges these exceptions or complications to medical records destruction but does not address practical mitigation strategies [8].

The process of adequately purging old studies may not be simple. To protect health information, our state requires destruction of the storage media or over-writing of the data, so that it cannot be recovered. It is impractical to destroy large storage systems if they may retain other needed studies. Further, over-writing is also a challenge as deletion of old studies may result in scattered re-usable storage blocks associated with fragmentation and compaction with potential impacts on retrieval time.

At a societal level, mass destruction of older images could be a potential loss for future clinical research, especially in this era of rapid evolution of image interpretation algorithms based upon artificial intelligence developed from massive clinical imaging archives. Other important considerations for deletion of old imaging tests include the following: imaging studies obtained for research purposes may be admixed in a clinical PACS and have different retention requirements; patients may have expectations for long-term retention of their images when seeking longitudinal care; and the risk of accidental deletion of desirable studies when executing mass deletion.

In conclusion, when considering the destruction of older imaging exams due to cost and privacy concerns, we should also be mindful of the potential clinical value to patients when we retain them and make them readily accessible. We found that our studies over 10 years of age were commonly called upon for viewing in the PACS and thus potentially contributed to medical decision-making. This provides evidence that at least some older digital images should be retained longer for clinical benefit. Given the paucity of evidence-based literature on this question, our findings were helpful to our institution, and they may also be so to others facing similar decisions to purge old studies or not. Considering our findings, leaders at our institution decided (1) to continue to retain old exams for the sake of our patients in spite of the state policy and (2) to lobby the state to soften or extend the disposition requirements of old medical imaging tests. There is no further resolution for our state or institution as of the time of this writing.

While we found that many old exams still have potential value to our patients, our results unfortunately do not provide the answer regarding when to destroy old exams, or if that should vary by institution, practice environment, or patient scenario. Some of our staff have suggested using lack of recent encounters as a potential criterion to start the clock on purging. However, when looking to the future, as interoperability and image sharing across systems continue to evolve, there is an increased likelihood that old images from one institution could benefit the patient even when seeking care at another. One thing is clear: more clinical evidence is needed to establish guidelines for the deletion of images to prevent lost benefit to our patients.

## Data Availability

The authors declare that they had full access to all of the data in this study, and the authors take complete responsibility for the integrity of the data and the accuracy of the data analysis.
